# Biotics and bacterial function: impact on gut and host health

**DOI:** 10.1093/ismejo/wrae226

**Published:** 2024-11-05

**Authors:** Anwar Kandari, Ma’en Al Odat, Fawaz Alzaid, Karen P Scott

**Affiliations:** Dasman Diabetes Institute, Al-Soor Street, Dasman, 15462, Kuwait; Ministry of Health, Sulaibkhat, Jamal Abdel Nasser Street, PO Box 5, 13001, Kuwait; Medical Laboratory Science, Mutah University, Mutah, Karak 61710, Jordan; Dasman Diabetes Institute, Al-Soor Street, Dasman, 15462, Kuwait; INSERM UMR-S1151, CNRS UMR-S8253, Université Paris Cité, Institut Necker Enfants Malades, Paris, France; Gut Microbiology Group, Rowett Institute, University of Aberdeen, Aberdeen AB25 2ZD, UK

**Keywords:** gut bacteria, gut microbiota, enteropathogens, prebiotics, probiotics, synbiotics, postbiotics

## Abstract

The human gut microbiota, the vast community of microbes inhabiting the gastrointestinal tract, plays a pivotal role in maintaining health. Bacteria are the most abundant organism, and the composition of bacterial communities is strongly influenced by diet. Gut bacteria can degrade complex dietary carbohydrates to produce bioactive compounds such as short-chain fatty acids. Such products influence health, by acting on systemic metabolism, or by virtue of anti-inflammatory or anti-carcinogenic properties. The composition of gut bacteria can be altered through overgrowth of enteropathogens (e.g. *Campylobacter*, *Salmonella* spp.), leading to dysbiosis of the gut ecosystem, with some species thriving under the altered conditions whereas others decline. Various “biotics” strategies, including prebiotics, probiotics, synbiotics, and postbiotics, contribute to re-establishing balance within the gut microbial ecosystem conferring health benefits. Prebiotics enhance growth of beneficial members of the resident microbial community and can thus prevent pathogen growth by competitive exclusion. Specific probiotics can actively inhibit the growth of pathogens, either through the production of bacteriocins or simply by reducing the gastrointestinal pH making conditions less favorable for pathogen growth. This review discusses the importance of a balanced gut ecosystem, and strategies to maintain it that contribute to human health.

## Introduction to the gut microbiota

The digestive tract is the most heavily colonized site of the human body, containing microorganisms including bacteria, archaea, fungal cells, and viruses, which together add up to ~100 trillion cells [1, 2]. The composition of the gut bacterial ecosystem varies for each individual and changes during different stages of life [3]. Overall, the human body hosts a complex and diverse bacterial ecosystem, the composition of which depends on a combination of factors: diet, medical treatments, age, geography, and environment [4].

Composition and density of the microbiota depends on the colonization site within the gastrointestinal tract (GIT). The large intestine, or colon, is the most heavily colonized region of the gut. The colon contains the largest bacterial community, predominately strict anaerobes, due to its slow transit time and the mildly acidic conditions [5]. It contains the most nutrients for bacteria [6], and has ideal conditions for bacterial growth such as pH and temperature, and fewer host secretions including digestive enzymes and bile salts than higher up in the intestinal tract [7]. The bacteria in the colon comprise hundreds of different species [8], the proportions of these species and their genera are variable, whereas the specific phyla present are relatively consistent between different individuals [8]. According to molecular sequencing methods, the baseline healthy human gut microbiota consists of around 59 bacterial genera and 109 species [9].

Gut bacteria have many important beneficial functions for human health. Many of these bacteria can degrade complex carbohydrates that cannot be degraded by human enzymes. The fermentation process produces short-chain fatty acids (SCFA); butyrate, acetate, and propionate which play vital roles in maintaining human health. Some of the benefits of these metabolites include functioning as an energy source for gut epithelial cells, controlling anorectic hormones involved in appetite, and possessing anti-carcinogenic and anti-inflammatory properties [5]. Additionally, gut bacteria encode enzymes that assist in the biosynthesis of essential metabolites, including amino acids and vitamins important for the host [10, 11].

Gut bacteria also employ several mechanisms to defend against pathogens, including producing bacteriocins, which are specific toxins that target other bacteria; altering gut pH by producing SCFA; consuming limited common nutrients; and stimulating host immunity by promoting mucosal barrier function [8, 12]. Moreover, there are direct interactions between gut bacteria and host immune cells, where commensal gut bacteria stimulate the activation of mucosal B cells and some T cell subsets, creating a protective immune barrier against pathogenic bacterial invasion [13].

## Impact of diet on gut bacteria and human health

The composition of gut microbiota changes over the host’s lifespan. Variation in composition in adults depends heavily on diet [14, 15]. The dietary components that significantly impact the bacterial ecosystem are the major macronutrients such as carbohydrates, fats, and proteins [16]. Certain bacterial species are associated with specific types of diet. For example, *Bacteroides* spp. are abundant in those consuming high-protein diets, whereas *Prevotella* spp. abundance is associated with diets rich in fibers [17].

Consumption of fiber-rich diets is generally expected to increase the quantity and the diversity of gut microbiota [18], because fiber is the main source of nutrients for most gut bacteria. Some reports postulate that species diversity is only influenced by diet to a limited extent [19]. This might be because some individuals are not accustomed to consuming sufficient fiber, and certain bacterial species are not present in their gut ecosystem.

Fiber and starch are important substrates that promote beneficial gut bacteria, with glucose, a key monosaccharide, serving as an essential energy source [20] and carbon provider for bacterial growth and nucleic acid synthesis [21]. Most glucose from non-resistant starch, sucrose, and simple sugars is absorbed in the small intestine and does not affect the bacteria in the lower gut much. However, an observational cross-sectional study (n = 180) revealed that consuming a diet rich in various monosaccharide sources, such as non-glucose monosaccharides, enhances the diversity of gut microbiota and their metabolites [22].

Gut bacteria encode many different types of carbohydrate-active enzymes responsible for utilization of specific carbohydrate substrates that enter the large intestine [23, 24]. These bacteria, in turn, produce different fermentation end-products according to species and carbohydrate intake. Different carbohydrates, composed of different monosaccharides, have varied effects because they alter bacterial composition by promoting the growth of different bacteria. For example, it has been found that buckwheat honey, which contains honey oligosaccharides, selectively enhances the growth of the indigenous beneficial bacterial genus *Bifidobacterium* compared to a control group of fructooligosaccharides (FOS) [25]. Another study indicated that the intake of various non-digestible carbohydrates results in lower inflammatory responses compared to a diet high in simple sugars such as glucose and fructose [22].

Human intervention studies frequently compare the impact of consuming different carbohydrates and fibers by measuring SCFA concentrations in fecal samples. However, these studies may produce inconsistent results. For instance, in one study, the consumption of a variety of fiber sources contained within whole foods did not significantly increase fecal SCFA concentrations [19], whereas many other human studies have shown that fibers increase SCFA production in the gut [26, 27]. Consumption of FOS and galactooligosaccharides was shown to reduce concentrations of total SCFA and acetate, and significantly decreased butyrate [28]. However, another study stated that FOS increased butyrate production by some bacterial species *in vitro* [29]. Such variation in results of human studies occurs because of several factors, including balance of SCFA production and utilization by gut bacteria, the absorption of SCFA by host epithelial cells, the effect of different diets on gut microbiota compositions, and the inter-individual variation in responses.

Fecal SCFA concentrations represent a balance of production, utilization, and absorption in a complex ecosystem, and care must be taken in extrapolating results to infer colonic concentrations. Moreover, it is challenging to directly compare the results of studies conducted with live human volunteers with those from preclinical or *in vitro* models, due to various factors such as limited carbon sources *in vitro*. For example, metabolic cross-feeding, or bacterial metabolic interactions, is a fundamental mechanism in the gut, where one bacterium produces metabolic end-products (including fermentation acids), and other bacterial species use these products for survival [30], often releasing gases and SCFA [31]. This mechanism is estimated to produce 20% of the total butyrate pool in the gut [30], and reduces the amount of lactate [32]. Concentrations can also be affected by differences in the amount of host epithelial tissue to absorb SCFA due to epithelial damage caused by inflammatory bowel diseases [33]. Consequently, SCFA may pass through the intestines without being absorbed, potentially affecting metabolic and immune function, and leading to unusually high fecal concentrations. These discrepancies highlight the need for more human studies to fully understand the dynamic process of SCFA production/utilization/absorption after carbohydrate consumption.

**Figure 1:**
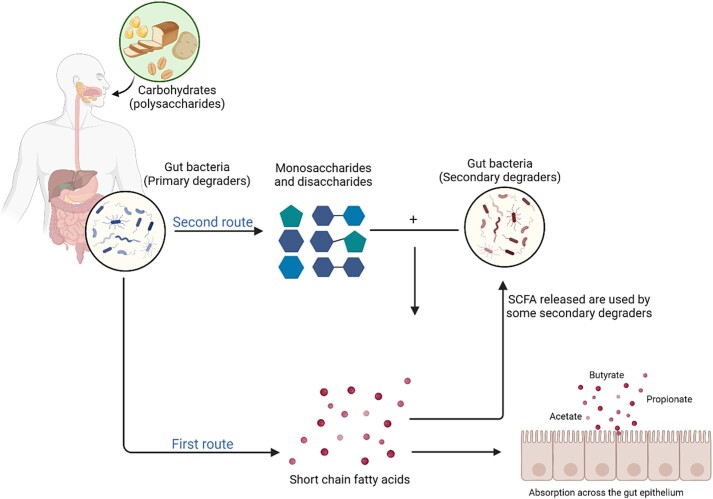
**The main routes of carbohydrate fermentation in the large intestine.** The first route involves the direct degradation of polysaccharides by primary degraders using either the glycolytic pathway or pentose phosphate pathway producing SCFA directly. The second illustrates degradation of polysaccharides into monosaccharides and disaccharides that are then utilized by secondary degrader bacteria to produce SCFA. The SCFA produced may then be absorbed by the epithelium cells in the gut and some then circulate around the body in the bloodstream. Based on Macfarlane and Macfarlane, 2003 [20] and Scott et al., 2008 [35]. Created with BioRender.com.

Gut bacteria utilize substrates under anoxic conditions in the large intestine. Monosaccharides can enter the glycolytic pathway to produce pyruvate and energy or the pentose phosphate pathway to generate NADPH and intermediates like fructose-6-phosphate and glyceraldehyde-3-phosphate, which subsequently enter glycolysis [20, 34].. The precise routes of carbohydrate degradation depend on specific gut bacterial enzymes present and the specific types of polysaccharides available [20, 34]. Some bacteria, known as primary degraders, break down complex polysaccharides, releasing monosaccharides, disaccharides, and oligosaccharides which may then be degraded by other gut bacteria (secondary degraders) to produce SCFA (Fig. 1). Excess hydrogen ions may be utilized directly thorough cross-feeding by one of three groups of hydrogenotrophic gut bacteria producing either hydrogen sulphide, methane, and acetate. Alternatively, hydrogen may enter the bloodstream and circulate around the body (Fig. 1) [35].

Although *Bacteroides* spp. increase during consumption of an animal-based diet, some bacteria within this genus have many genes encoding plant cell wall degradation enzymes (glycan-cleaving enzymes) and can utilize many different polysaccharides. Thus, *Bacteroides* spp. might be primary degraders for many complex carbohydrates [36]. In contrast, *Ruminococcus* spp., especially *Ruminococcus flavefaciens*, can degrade plant cell wall components such as cellulose and pectin [37] because they secrete hydrolytic enzymes [38]. Depending on the bacterial species present and the availability of specific carbon sources in the large intestine, the proportion and the types of fermentation products will vary although specific SCFA, hydrogen, carbon dioxide, and adenosine triphosphate are consistently found [39, 40].

## Bacterial fermentation products and human health

Microbial fermentation products may be divided into three main groups. Firstly, SCFA which include butyrate, acetate, valerate, propionate, and caproate, are formed from the fermentation of carbohydrates and/or amino acids. Secondly, branched chain fatty acids (BCFA) consist of iso-butyrate, iso-valerate, and 2-methyl-butyrate, and these acids are formed during the catabolism of branched-chain amino acids such as valine, leucine, iso-leucine [20]. The third group comprises other bacterial fermentation products such as lactate, ethanol, succinate, and gases. The major metabolites produced in the colon are acetate, propionate, and butyrate in the ratio of 3:1:1 [41], whereas valerate, caproate, formate, and the BCFAs iso-butyrate, 2-methyl-butyrate, and iso-valerate are produced in lower amounts [20].

These fatty acids play a vital role in modulating intestinal structure and function, as well as contributing to overall host health. For instance, BCFA may offer protective effects against metabolic risk factors [42]. *In vitro* studies showed that exposing Caco-2 cells, a model of the intestinal barrier, to BCFA reduces the activity of proinflammatory markers, such as IL-8 and NF-κB [43]. Additionally, specific BCFA (iso-14:0, iso-16:0, and anteiso-13:0) improve the viability of cells affected by lipopolysaccharides. Systemic reviews indicate that elevated circulating BCFA levels are associated with improved cardiometabolic health, including better regulation of blood lipids, enhanced insulin sensitivity, and a reduced risk of developing conditions such as type 2 diabetes and cardiovascular diseases [42]. However, individuals with hypercholesterolemia exhibit higher fecal levels of BCFA, particularly iso-butyric acid, which correlate with a more adverse lipid profile in serum [44]. In contrast, BCFA can stimulate hepatic gluconeogenesis, potentially elevating blood glucose levels and exacerbating metabolic conditions [45]. Despite these findings, research on BCFA production and its health effects remains limited and often inconsistent, largely due to a predominated focus on protein intake and dietary supplement interventions.

SCFA are particularly vital for maintaining overall host health, having anti-carcinogenic and anti-inflammatory properties [5]. Several factors affect the types and amounts of these metabolites produced in the colon, including bacterial composition, presence of nutritional substances for bacteria in the diet [46], and the length of time fermentable materials spend in the colon.

The consumption of high-fiber diets and the production of SCFA can reduce lipid concentrations in blood, thus decreasing the risk of cardiovascular disease [47–50]. Trace amounts of SCFA move from the intestine into the bloodstream and are metabolized in various locations around the body. Most butyrate remains in the colon where it is an important energy source for gut epithelial cells [51]. An abundance of butyrate has been associated in the prevention and treatment of inflammatory bowel disease and colorectal cancer [51, 52]. Another study stated that butyrate promotes apoptosis in cancer cells by regulating gene expression in the colon epithelial cells and reducing tumor formation [53]. The anti-inflammatory role of butyrate is by inhibiting NF-KB and histone deacetylase, which controls expression of inflammatory effectors and reduces migration of neutrophils by preventing the production of cytokines and chemokines at the location of inflammation [54]. The presence of butyrate in the liver reduces insulin resistance and obesity; specifically, when the concentration of plasma butyrate increases, blood lipids are decreased [55]. In a study of 60 heart failure patients, lower butyrate levels in these patients were found to be indicative of small intestinal bacterial overgrowth. The study suggests that butyrate deficiency may contribute to increased gut permeability, triggering inflammation and exacerbating heart failure [56]. Oral administration of butyrate significantly inhibited high-fat diet-induced atherosclerosis and hepatic steatosis in Apolipoprotein E-deficient mice and modulated expression of genes related to lipid and glucose metabolism in the liver [57].

Acetate, produced by a wide variety of gut anaerobic bacteria, is the most abundant SCFA found in the human large intestine. It is used for cholesterol synthesis and is also widely utilized by other gut bacteria to produce butyrate and other SCFA products [58]. Additionally, acetate increases blood flow and oxygen uptake in the colon, it improves ileal movement by influencing ileal contraction [59], and enhances the function of the gut epithelium barrier [60]. An *in vitro* study indicated that higher circulating acetate levels were correlated with reduced visceral fat, suggesting acetate’s beneficial role in mitigating cardio-metabolic disease risk by influencing fat storage [61]. Another study found that acetate in mouse models enhances the host’s antiviral response against influenza A virus infection, providing protective effects [62].

Propionate is also known to offer numerous benefits for human health, such as lowering cholesterol, anti-inflammatory, anti-lipogenic, anti-carcinogenic [59, 63], and enhancing satiety [64]. Propionate’s anti-tumor effects in colorectal cancer are mediated by upregulating apoptosis-related genes (such as *HECTD2*) [65]. Its presence in the liver reduces liver triglyceride and plasma phospholipid concentrations, resulting in reduced liver steatosis [66]. Similarly to butyrate, propionate acts as gluconeogenic substrate, which decreases lipid and glucose metabolism and reduces metabolic disturbances related to obesity [66]. Furthermore, propionate can decrease cholesterol biosynthesis in the liver by inhibiting 3-hydroxy-3-methylglutaryl-CoA reductase (*HMGCR*) [49], the enzyme responsible for cholesterol metabolism. Increasing propionate production consequently reduces low-density lipoprotein (LDL) cholesterol. In addition, an *in vitro* study showed that propionate-producing bacteria improve insulin resistance and reduce liver inflammation by influencing gut-liver communication, as propionate enhances glycogen storage in hepatic cells and reduces expression of pro-inflammatory markers [67]. Gut bacteria and their metabolites also support human health by influencing similar pathways involved in immune function and inflammatory bowel diseases.

## Impact of enteropathogens on the gut microbiota

The presence of pathogenic bacteria in the gut can disrupt the composition of the diverse range of beneficial bacteria normally present in the human gut and lead to various health problems. Infections caused by enteropathogens are typically associated with symptoms such as diarrhea, abdominal pain, fever, and vomiting. Diarrhea in children may be caused either by rotavirus infection or by several different pathogenic bacteria including *Shigella* spp., *Campylobacter* spp., *Salmonella* spp., *Streptococcus* spp., *Escherichia* spp., and *Plesiomonas* spp. [68–72].

The many pathogenic bacteria that have the potential to cause gastrointestinal infections often disrupt the gut microbiota by outcompeting commensal bacteria, producing toxins, and inducing inflammation in the gut [73]. The resulting microbiota imbalance causes gastrointestinal symptoms and may lead to more severe conditions, such as potential carcinogenicity and tumor promotion [74], whereas any invasive enteropathogens entering the bloodstream can cause septiceamia [75].

*Salmonella* and *Campylobacter* are two distinct genera of bacteria that are commonly associated with foodborne illnesses, sharing some similarities in gastrointestinal disease symptoms but also have notable differences. *Salmonella*, a genus of Gram-negative bacteria within the family of Enterobacteriaceae [76, 77] includes various species, encompassing more than 2500 serovars in animals, humans, and environments [78]. *Salmonella* infections, known as salmonellosis, are a major cause of foodborne infections globally, leading to substantial morbidity. Person-to-person transmission also occurs, particularly between patients and/or in unsanitary environments [79, 80]. The pathogenicity of *Salmonella* spp. stems from their ability to invade host cells. Key virulence factors include the Type III secretion system (T3SS), allow the bacterium to inject toxins into host cells, disrupting cellular metabolism [81]. Bacteria of this genus can colonize and persist in the GIT of humans [82] and induce acute intestinal inflammatory responses, enhancing their transmission and growth in the GIT [83]. *Salmonella enterica* interacts with the host through various mechanisms by possessing factors such as secretion systems, flagella, fimbriae, endotoxins, and exotoxins which play critical roles in successfully infecting host cells [84].

*Campylobacter* is another genus of Gram-negative bacteria that includes several species causing gastrointestinal infections in humans. The most common infectious species is *Campylobacter jejuni*, followed by *Campylobacter coli* [85]. These bacteria are a leading cause of bacterial foodborne illness (campylobacteriosis) worldwide, causing more infections than *Salmonella* spp., and often result from the consumption of contaminated poultry and unpasteurized milk [86]. Infections with *Campylobacter* spp. typically lead to symptoms such as diarrhea, blood in stool, abdominal pain, fever, and sometimes nausea and vomiting, and these illnesses usually last for a few days to a week [73].

**Figure 2:**
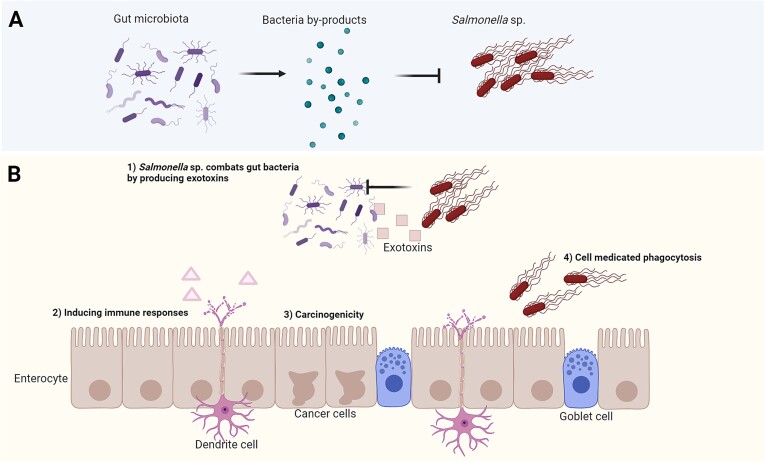
**Interaction between *salmonella* sp. and the intestinal microbiota. (**A) Colonization resistance is facilitated through the production of metabolites by the gut microbiota to inhibit the invasion of pathogens such as *salmonella* sp. (B) Four mechanisms through which *salmonella* sp. evades colonization resistance. (1) direct competition between *salmonella* sp. and the normal commensal bacteria by enterotoxin production. (2) infection induces an immune response which negatively impacts the colonization of beneficial bacteria. (3) *salmonella* sp. affects enterocytes resulting in growth of tumor cells. (4) host cell mediated phagocytosis induced by injecting enterotoxins in both goblet cells and enterocytes via T3SS, helping *salmonella* sp. avoid the immune response. Based on Ahmer and Gunn, 2011 [88] and Thiennimitr et al., 2011 [89]. Created with BioRender.com.

In most instances, incoming microorganisms typically cannot establish themselves in a healthy gut and disturb the balance of the gut microbiota [87]. Under certain conditions, however, *Salmonella* spp. can overcome host colonization resistance by triggering localized inflammation. When phagocytic cells engulf *Salmonella* cells, it leads to host cell signaling that induces innate resistance mechanisms. One consequence is the release of more growth substrates some of which *Salmonella* spp. utilize effectively, outcompeting the resident microbiota [88] (Fig. 2). For instance, *Salmonella* spp. can utilize ethanolamine as a nutrient source, enabling proliferation and establishment within the inflamed intestinal environment [89]. Additionally, *Salmonella* spp. can compete with commensal microbiota for attachment sites on intestinal epithelial cells further disrupting the equilibrium of the gut microbiota. Infections by *Campylobacter* spp. also alter the composition of the gut microbiota. After campylobacteriosis, a decrease in bacteria within the Lachnospiraceae family, particularly *Dorea* and *Coprococcus* spp., was observed, whereas increased levels of *Bacteroides* and *Escherichia* spp. were present in the gut microbiota [90]. The colonization of *Campylobacter* spp. in poultry abattoir workers led to a persistent long-term alteration in their gut microbiome [91].

Numerous studies have been conducted to examine how pathogenic bacteria affect human health (Table 1). These studies are vital for understanding the mechanisms of infection, the impact on the human body, and for developing strategies to prevent and treat such infections. For example, a study was conducted to determine the impact of *Campylobacter* spp., *Salmonella* spp., Shiga toxin-producing *Escherichia coli*, and *Shigella* spp. on the gut microbiota by comparing the fecal microbiota in 200 individuals with diarrhea in comparison to 75 healthy volunteers [92]. The results of the study showed that *Escherichia* spp. predominated in the gut of diarrheal patients, while beneficial genera including *Bacteroides*, *Prevotella*, *Roseburia*, *Lachnospira*, and *Blautia* ssp. were more prevalent in the healthy volunteers [92]. Another study investigated the alteration of gut microbiota composition in nine children suffering from acute watery diarrhea due to cholera. The primary phylum observed in cholera cases was Proteobacteria particularly pathogenic bacterial genera, with concomitant reductions of major commensal bacteria belonging to the Firmicutes, Bacteroidetes, and Actinobacteria phyla [93]. The gut microbiota composition of 508 diarrheal children infected with *Escherichia/Shigella, Granulicatella,* and *Streptococcus mitis/pneumoniae* revealed a reduction in *Lactobacillus ruminis* and an increase in *Lactobacillus, Neisseria, Citrobacter, Erwinia*, and *Haemophilus.* The same study observed that the gut bacterial composition of 484 healthy children contained more obligately anaerobic bacteria such as *Bacteroides fragilis, Dialister* spp.*, Megasphaera* spp.*, Mitsuokella/Selenomonas* spp.*, Prevotella* spp., and *Clostridium difficile* [72]*.* In another study involving 32 diarrheal children infected with *Escherichia coli*, there was a decrease in Firmicutes and an associated increase in the phyla Proteobacteria and Bacteroidetes, with specific increases in the genera *Pseudocitrobacter*, *Bacteroides*, and *Escherichia-Shigella* compared to 30 healthy children [94]. *Bacteroides* is a diverse genus, with many species a major part of the healthy microbiota, whereas some are opportunistic pathogens, and other produce specific toxins [95].

**Table 1:** Summary of peer-reviewed studies on microbiological profiles.

Study		Number of participants	Diarrheal etiology	Study method	Increased	Decreased
Singh et al., 2015 [92]	Diarrheal patients	200	*Campylobacter, Salmonella*, Shiga toxin-producing *E. coli* and *Shigella*	16S rRNA gene amplicon sequencing	**Phylum:** Proteobacteria **Family:***Enterobacteriaceae, Pateurellaceae, Lactobacillales* and *Streptococcaceae* **Genera:***Escherichia*	N/A
	Healthy participant	75	-		**Phylum:** Bacteroidetes and Firmicutes **Family:***Rikenellaceae, Bacteroidaceae, Ruminococcaceae, Porphyromonadaceae, Bifidobacteriaceae, Alcaligenaceae, Odoribacteraceae, Barnesiellaceae*, and *Lachnospiraceae*. **Genera:***Bacteroides, Prevotella, Roseburia, Lachnospira*, and *Blautia*	
Monira et al., 2013 [93]	Diarrheal patients	9	*Vibrio cholera*	16S rRNA gene amplicon sequencing	**Phylum:** Proteobacteria **Families**: *Vibrionaceae, Enterobacteriaceae, Prevotellaceae, Enterobacteriaceae, Prevotellaceae, Actinomycetaceae, Mycoplasmataceae, Streptococcaceae, Veillonellaceae* *Bacteroidaceae, Bifidobacteriaceae, Ruminococcaceae* *Enterococcaceae, Veillonellaceae* and *Enterobacteriaceae*	**Phylum:** *Firmicutes, Bacteroidetes,* and *Actinobacteria*
Pop et al., 2014 [72]	Diarrheal patients	508	*Escherichia/Shigella, Granulicatella* and *Streptococcus mitis* and *Streptococcus pneumoniae*	16S rRNA gene amplicon sequencing	**Genera:** *Lactobacillus, Neisseria, Citrobacter, Erwinia*, and *Haemophilus*	**Species:** *Lactobacillus ruminis*
	Healthy participant	484	-		**Family:** *Peptostreptococcaceae, Eubacteriaceae*, and *Erysipelotrichaceae* **Genera:***Clostridium sensu stricto, Dialister, Enterococcus, Megasphaera, Mitsuokella, Prevotella, Ruminococcus, Selenomonas* and *Turicibacter* **Species:***Bacteroides fragilis,* and *Clostridium difficile.*	
Gallardo et al., 2017 [94]	Diarrheal patients	32	Diarrheagenic *Escherichia coli*	16S rRNA gene amplicon sequencing	**Phylum:** Proteobacteria, Bacteroidetes **Families:***Enterobacteriaceae* and *Bacteroidaceae* **Genera:***Pseudocitrobacter, Bacteroides* and *Escherichia-Shigella* **Species:***Escherichia albertii, Citrobacter werkmanii, Yersinia enterocolitica* subsp. *paleartica*, and *Haemophilus sputorum*	
	Healthy participant	30	-		**Phylum:** Firmicutes **Families:***Erysipelotrichaceae*	

These examples show that it is critical to manage and restore the balance of the gut microbiota after infections with different pathogens. One opportunity exists in utilizing probiotics and prebiotics due to their established roles in maintaining a healthy gut ecosystem.

## Concept of ‘biotics

**Figure 3:**
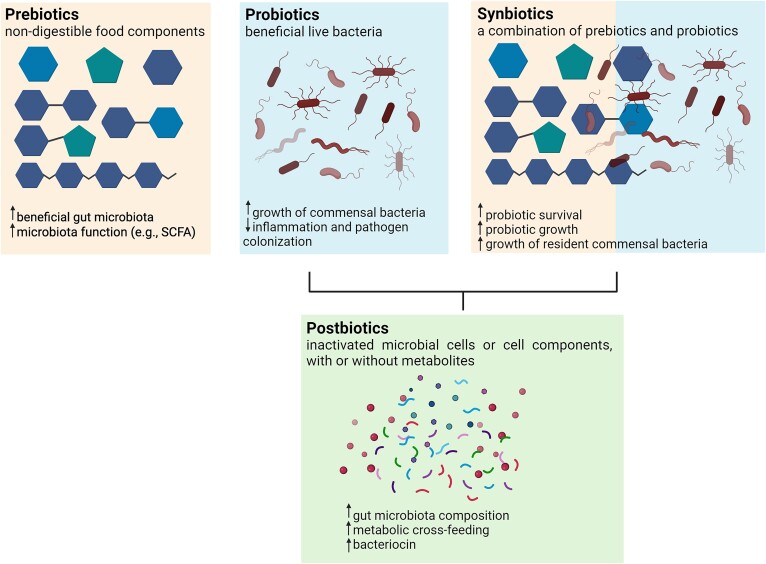
**Overview of some of the mechanisms through which ‘biotics act in the large intestine to improve host health.** Prebiotics are specific nutrients for resident gut bacteria, and promote the proliferation of specific beneficial gut bacteria, producing to SCFA and other health-promoting metabolites. Probiotics are live bacteria that promote a healthy gut ecosystem and confer various health benefits to the host. Synbiotics combine live bacteria and substrates in a synergistic or complementary manner to enhance the growth and activity of beneficial gut bacteria, thereby promoting digestive health and well-being. Postbiotics aim to harness the health-promoting properties of inactivated microbial cells or their metabolic byproducts to positively influence the host’s health. Based on Gibson et al., 2017 [98], Hill et al., 2014 [111], Gareau et al., 2010 [113], Butel et al., 2014 [114], Swanson et al., 2020 [152], Sergeev et al., 2020 [96], Salminen et al., 2021 [159], and Peluzio et al., 2021 [161]. Created with BioRender.com.

‘Biotics encompass a spectrum of substances and processes involving living organisms that profoundly influence health, with the focus here on gut health. Each of probiotics, prebiotic and synbiotics can play a vital role in maintaining a balanced bacterial ecosystem contributing to health (Fig. 3). Additionally, postbiotics, metabolic products of bacterial fermentation, can also exert beneficial effects on the host (Fig. 3). There is a general intention to improve health by enhancing the beneficial gut ecosystem through consuming supplements or foods enriched with such ‘biotics [96].

### Prebiotics and the gut microbial ecosystem

The term prebiotic was originally defined as “a non-digestible food ingredient that beneficially affects the host by selectively stimulating the growth and/or activity of one or a limited number of bacteria in the colon and thus improves host health” [97]. This term was updated in 2017 to “a substrate that is selectively utilized by host microorganisms conferring a health benefit” [98]. There are numerous compounds proposed as prebiotics, including peptides, polyphenols, lipids, proteins, and other non-digestible carbohydrates [99]. However, the term prebiotic historically refers to specific carbohydrates often short-chain oligosaccharide carbohydrates [100]. Dietary substrates considered as potential prebiotics have different chemical structures, physical states, and physiochemical properties depending on their sources [101], but are all non-digestible food components [97]. There are four specific criteria required to consider dietary substrates as prebiotics: the prebiotic should not be hydrolyzed by human enzymes and absorbed within the upper GIT, it should be selective for certain beneficial gut bacteria, it must alter the composition and/or function of those beneficial gut bacteria, and it should induce beneficial systemic effects to host health [97, 102, 103]. Most established prebiotics were selected to enhance the growth of lactic acid producing bacteria [104], therefore, there is potential to develop new prebiotics to selectively promote the growth and/or function of other beneficial gut bacteria to increase fermentation acid production and provide other health benefits.

Dietary carbohydrates are divided into digestible and non-digestible carbohydrates, with only digestible carbohydrates being broken down by human enzymes and subsequently absorbed in the upper GIT [105]. Complex, non-digestible carbohydrates cannot be broken down by the human digestive system due to a lack of enzymes responsible for breaking down specific bonds between the sugar units [106]. Non-digestible carbohydrates with high and medium molecular weights are broken down into low molecular weight substrates by specific bacterial carbohydrate-active enzymes.

Currently, there is increasing awareness that consuming dietary fiber from different sources has important, different health benefits for humans. The benefits of cereal grain fibers include the increased production of bacterial metabolites, especially SCFA, in the gut, which help regulate glucose metabolism and reduce the risk of type 2 diabetes [107], and reduce LDL cholesterol [108]. Consuming a diet rich in resistant starch also reduced fasting glucose in overweight adults by altering the gut microbiota composition [109]. Another study reported that high doses of oligofructose-enriched inulin, an fructan-type prebiotic, decreased the severity of ulcerative colitis in patients by altering the mucosa-associated microbiota and increasing fecal total SCFA and butyrate production [110]. Consequently, the effect of distinct dietary fibers, or specific prebiotics, on the composition of the gut microbiota and metabolites production should be measured to ascertain the beneficial effects of these substrates on human health.

### Probiotics and the gut microbial ecosystem

The World Health Organization (2001) initially defined probiotics as “live microorganisms which when administered in adequate amounts confer a health benefit on the host”. However, many products labelled as probiotics often do not meet probiotic criteria such as defined contents, appropriate viable count at end of shelf-life, and proper evidence for health benefits. The accepted, updated definition in 2014 describes probiotics as “live microorganisms that when administrated in adequate amounts confer a health benefit on the host”, and the paper includes additional specification that they should be “products that deliver live microorganisms with a suitable viable count of well-defined strains with a reasonable expectation of delivering benefits for the wellbeing of the host” [111]. The European Food Safety Authority (EFSA) regulates new food products in Europe, and companies must submit dossiers of evidence demonstrating health benefits for any products they wish to market as probiotics. Similarly, after nearly five years of evaluation, the United States Food and Drug Administration has determined that there is credible evidence to a health claim stating that regular consumption of yogurt made from milk fermented with *Lactobacillus bulgaricus* and *Streptococcus thermophilus* may reduce the risk of type 2 diabetes. This decision is based on limited but credible scientific evidence and marks the first time such a claim has been approved for yogurt [112]. Although many live bacteria may be present in foods and supplements, only a few specific strains with scientifically proven health benefits can be officially called probiotics.

Renewed interest in probiotics among scientists and food manufactures is driven by consumer awareness of their positive physiological effects, particularly on the GIT. Bacteria currently used as probiotics are thoroughly tested for their efficiency and safety. Probiotics have a variety of actions within the host dependent on the bacterial strains’ specificity and mechanisms of action [113]. Probiotics can interact with the gut microbial community to prevent or limit pathogen colonization, produce fermentation acids, and slightly lower the GIT pH for favorable bacterial growth [114]. *Bifidobacterium* spp. were first isolated by Henry Tissier in 1899 [115, 116], and he was the first scientist to investigate the role of the beneficial bacteria in treating intestinal diseases [115]. In contrast, Elie Metchnikoff (Metchnikov) demonstrated the role of *Lactobacillus* spp. for health in 1907, and he is known as the grandfather of modern probiotics [117].

Most probiotics marketed in food products belong to *Lactobacillus* and *Bifidobacterium* genera [118, 119], with only a few other species in products. Both *Lactobacillus* and *Bifidobacterium* genera occur naturally in the intestine, are known to be non-pathogenic, and large-scale manufacture is relatively easy. Various health benefits have been attributed to representative species of these bacteria. For instance, consuming yogurt enriched with *Lactobacillus* spp. reduces the duration of diarrhea [120], and decreases lactose intolerance symptoms [121–123], due to the ability of these bacteria to digest lactose [124]. *Lactobacillus* spp. survive well in the harsh conditions of the gut, tolerating the gastric fluids and low pH, and they produce fermentation acids [125]. *Bifidobacterium* spp. have various features making them useful as probiotics, including resisting bile salt, acidic conditions, and pancreatic enzymes and they can also associate with intestinal cells [126]. They can utilize a variety of dietary carbohydrates for growth, producing significant amounts of lactate and acetate, they induce anti-inflammatory effects, and prevent infection, particularly of some strains of pathogenic *E. coli* [60]. One study indicated that *Bifidobacterium* spp. supplements decreased lipid levels and cholesterol in children with hypercholesterolemia and hypertriacylglycerolemia by reducing LDL cholesterol [127], due to their bile salt hydrolase activity [128]. The two genera have different abilities to metabolize different carbohydrates in the gut; e.g. *Bifidobacterium* spp. can utilize fructose 6-phosphate, whereas *Lactobacillus* spp. can utilize glucose 6-phosphate, due to encoding specific enzymes for utilizing different carbohydrates [129, 130]. Many studies now focus on combining several probiotic bacterial genera to improve their mechanisms of action in the human gut [131].

Novel bacterial strains are being introduced and tested as probiotics for treating various diseases and disorders. For example, gavage of *B. fragilis* regulated butyrate metabolism and restored the response to methotrexate, a drug that modified disease progression in the treatment of rheumatoid arthritis, in gut microbiota-deficient mice. Suggesting new strategies to enhance methotrexate treatment in rheumatoid arthritis [132]. However, another murine study reported that *Bacillus fragilis* along with a high-fat diet increased food intake, elevated fasting blood glucose, body weight, and low-density lipoprotein levels, and reduced the abundance of *Lactobacillaceae* in mice [133]. Consumption of a cocktail of *Bacillus* spp. (*Bacillus subtilis* DE111®, *Bacillus megaterium* MIT411, *Bacillus coagulans* CGI314 and *Bacillus clausii* CSI08) over a period of 45 days significantly reduced loose stool in individuals, with no observed side effects, indicating that regular consumption at dose 2 × 10^9^ CFU/day is safe and well tolerated in healthy individuals [134]. *Akkermansia muciniphila,* recognized as “a potential next-generation beneficial microorganism” due to its beneficial role in host health [135]. It plays a key role in improving metabolic disorders, such as obesity [136] and diabetes [137], as well as maintaining gut health [138], regulating immunity, and reducing inflammation [139]. For example, supplementation with *A. muciniphila* in overweight and obese individuals (n = 40) slightly reduced body weight, fat mass, and decreased in blood markers associated with liver dysfunction and inflammation [136].

Expanding the focus beyond traditional probiotic strains such as *Bifidobacterium* and *Lactobacillus*, the potential use of other bacterial strains is gaining interest. The process of identifying and developing potential probiotics involves multiple stages, including the isolation and identification of bacterial strains, taxonomic classification through genetic and phenotypic methods, and the characterization and evaluation of their properties [140]. These could present challenges in developing new probiotics. Another challenge is that many probiotic strains are sensitive to oxygen concentrations which can compromise their viability during storage and transportation. This issue can be mitigated by optimizing cultivation methods. For example, the strictly anaerobic bacterium *Faecalibacterium prausnitzii* is highly sensitive to oxygen. It has been tested as a potential probiotic by co-culturing with *Desulfovibrio piger*, a sulfate-reducing bacterium. This symbiotic relationship, combined with the presence of the antioxidant cysteine, enables the production of *Faecalibacterium prausnitzii* in sufficient amounts for human administration [141].

Novel genetically modified bacterial strains have also been tested as probiotics due to their potential to rectify enzyme deficiencies in humans [142], or to target a specific pathogen or toxin in the host [143]. However, genetically modified food or food supplements are not accepted worldwide due to concerns about their safety, and they currently have limited clinical application [144]. Nowadays, much research is focused on identifying novel bacteria to be developed as new bacteriotherapy for additional diseases [145]. These new probiotics, sometimes referred to as “next-generation probiotics” [146] are being identified from the natural gut bacteria population, often due to the absence of specific microorganisms in individuals with certain diseases [147, 148].

Probiotics are generally consumed to enhance health, yet they can also cause side effects. Some individuals may experience bloating or diarrhea due to alterations in gut microbiota, leading to increased gas and acid production. These symptoms might last for a few days, but they can persist longer in some cases. Additionally, probiotics that are beneficial in healthy individuals could potentially become harmful in those with intestinal inflammation. For example, increased levels of *Bifidobacterium* and *Lactobacillus* have been observed in patients with active inflammatory bowel disease, possibly due to the reduction of butyrate-producing bacteria [149]. This imbalance may result from the high colonization of these probiotics, which can outcompete other beneficial gut bacteria. Another concern is that some probiotics may become pathogenic. For instance, *Lactobacillus spp.*, can become pathogenic in immunocompromised individuals [150]. Moreover, at least five several case reports have documented the occurrence of bacteremia in hospitalized patients following the use of *Clostridium butyricum* MIYAIRI 588 as a probiotic [151]. Therefore, careful consideration and appropriate prescribing practices are essential to minimize these risks and ensure individual safety.

### Synbiotics and the gut microbial ecosystem

The purposeful combination of probiotics and prebiotics is known as synbiotics. According to the latest definition from the International Scientific Association for Probiotics and Prebiotics (ISAPP), a synbiotic is “a mixture comprising live microorganisms and substrate(s) selectively utilized by host microorganisms that confers a health benefit on the host” [152]. This definition means that components not themselves proven probiotics or prebiotics can be incorporated into synbiotics, as long as the combined product meets the rest of the criteria [152]. The term synbiotic is further sub-divided into two categories: complementary synbiotic and synergistic synbiotics. Complementary synbiotics contain a substrate and live bacterium that together confer additional health benefits although they act independently, and both independently meet the current definition of prebiotic or probiotic. A synergistic synbiotic contains a substrate that is selectively utilized by the bacterial component [98], and may also be used by the resident microbiota. Both types of synbiotics have different mechanisms by which they exert their effects on the microbial ecosystem. One aim of synbiotics, particularly synergistic synbiotics, is to stimulate the growth of the probiotic bacterium and enhance probiotic survival during passage through the GIT [153].

The health benefits of synbiotics should be greater than using either component individually. For instance, consumption of a synbiotic containing galactooligosaccharides (GOS) and lactic acid bacteria (*Lactobacillus acidophilus*, *Bifidobacterium lactis*, *Bifidobacterium longum*, and *Bifidobacterium bifidum*) increased the abundance of beneficial gut bacteria, including *Ruminococcus* spp., *Bifidobacterium* spp., *Lactobacillus* spp., *Coprococcus* spp. [96]. A synbiotic combination of *B. lactis*, *Lactobacillus casei*, and *Lactobacillus plantarum* together with crystalline cellulose and lactose improved bowel health and changed the composition of the gut microbiota after three weeks consumption, with the abundance of some beneficial genera such as *Bifidobacterium*, *Faecalibacterium*, and *Fusicatenibacter* increasing compared to the baseline [154]. Research on healthy postmenopausal women revealed that synbiotic fermented milk, containing *Lactobacillus paracasei* and inulin, improved the oral bioavailability and plasma levels of isoflavones [155], which are crucial for promoting heart health [156]. The consumption of freeze-dried *B. longum* in combination with Synergy 1 (FOS/inulin mix) for 1 month by 18 patients with active ulcerative colitis showed a reduction in the mucosal inflammation of the intestine and a reduction in colitis. This therapeutic effect was attributed to the adhesion of the bacteria to epithelial cells and their abilities to utilize the prebiotic as an energy source [157]. In an animal study, consuming a synbiotic mixture of *Lactobacillus bulgaricus*, inulin, and FOS for 16 weeks increased the production of fermentation acids and, in turn, led to a reduction in the indoxyl sulphate, which then reduced the development of chronic kidney disease in mice [158]. Therefore, synbiotics are becoming widely used to exert their beneficial effects in numerous health conditions and are now often prepared as supplements or additives in functional foods.

### Postbiotics and the gut microbial ecosystem

Recent findings have revealed that certain mechanisms through which bacteria promote health benefits are independent of cell viability. This has resulted in the emergence of terms such as postbiotic, alternatively known as paraprobiotic or metabiotic, to indicate that the bioactivity of non-viable microbial cells, microbial components, or cell lysates might potentially offer physiological benefits to the host. In 2019, ISAPP proposed the definition of postbiotics: “preparation of inanimate microorganisms and/or their components that confers a health benefit on the host” [159]. Thus, postbiotics are mixtures of bacterial components released after bacterial breakdown including structural molecules and potentially metabolic products such as SCFA, peptides, amino acids, some simple carbohydrates, and vitamins [160–162]. Applying the ISAPP definition [159], purified components like individual SCFA would not be postbiotics but would be specifically named, e.g. butyric acid. However other publications do refer to purified microbial products as postbiotics [163, 164].

The main aim of postbiotics is to improve health through promoting commensal bacterial growth or mirroring commensal bacterial activity. For example, metabolites produced by two species of *Lactobacillus*; *Lactobacillus fermentum*, and *Lactobacillus paracasei*, cultured with rice bran as prebiotics, suppress the growth of *Salmonella typhimurium* [165]. Bacteriocin metabolites produced by *Enterococcus faecalis* inhibit the activity of *Clostridioides difficile* [166]. Thus, postbiotics could serve as potential alternatives or supplementary treatments to using live microorganisms, although further studies are needed to demonstrate their clinical effectiveness. Bacterial metabolic cross-feeding, through which bacteria utilize the fermentation products of different bacterial species for growth [167], is certainly one mechanism for the action of postbiotics. As composition of the gut microbiota differs between populations and individuals, the impact of an added component exerting an activity through action of the microbiota, including postbiotics, varies between individuals. Evidence regarding the effect of postbiotics in modulating gut bacteria and their impact on human health is limited, although there are a few studies in animal models. For example, heat-inactivated *Lactobacillus gasseri* CP2305 improved stress-associated symptoms and the intestinal environment of mice [168]. A food supplement containing stabilized non-viable *Lactobacilli* fermentation product, enhanced the health, performance, immunity, and gut condition of broiler chickens even when challenged with *E. coli* [169]. The supernatants of specific *Bifidobacterium* strains, particularly *Bifidobacterium adolescentis*, inhibited the effect of *Candida albicans* infection *in vitro* due to fermentation acid production and the resulting acidic pH [170].

Various evidence suggests that pasteurization of *A. muciniphila* enhances its stability and, more notably, boosts its effectiveness [171]. In murine studies, an administration of pasteurized *A. muciniphila*, alongside a high-fat diet, reduced diet-induced obesity by downregulating lipid-droplet regulatory proteins linked to obesity [172, 173]. This intervention also improved energy expenditure, oxygen consumption, and physical activity, and increased fecal energy content and reducing the expression of carbohydrate transporters in high-fat diet-fed mice [172]. These findings position pasteurized *A. muciniphila* as a leading candidate among next-generation postbiotics. In 2021, the EFSA Panel on Nutrition, Novel Foods, and Food Allergens concluded that pasteurized *A. muciniphila* is safe at a dose of 3.4 × 10^10^ cells per day, provided that the number of viable cells remain below 10 cells per gram of the novel food [174].

Postbiotics are being introduced as an alternative to probiotics due to their ability to exert health effects independently of cell viability. The primary benefits of using postbiotics include enhanced safety due to the use of inactive bacteria and improved stability, as they do not interact with food components, thereby avoiding any impact on food quality [175]. Postbiotics incorporated into food products or used as food supplements potentially have easier manufacture and improved storage, handling, transportation, and shelf life compared to probiotics and thus have potential for future therapeutic applications. Unlike pharmaceuticals or biological products, postbiotics are generally regulated as dietary supplements.

## ‘Biotics in the defense against enteropathogens

‘Biotic therapies are increasingly recognized for their potential in combating pathogenic infections. These therapies operate through various mechanisms to enhance gut health and inhibit the growth of harmful bacteria. Various published studies have investigated the efficacy of probiotics and prebiotics in countering pathogenic bacteria (Table 2). These studies demonstrate that probiotics can inhibit pathogen colonization by employing competitive exclusion and producing antimicrobial agents and have been applied to both humans [176–186] and animals [166, 167]. Meanwhile, prebiotics, by promoting the selective growth of beneficial gut microbiota, strengthen the gut’s defenses against pathogenic invasion [189–191]. The clinical trials investigated the effects of probiotics on human diseases. In contrast, much of the research on prebiotics has been concentrated on animal models [189–191], and confirmatory studies in the target host would be required.

**Table 2:** Publications on the efficacy of probiotics and prebiotics in treating gastrointestinal pathogens.

Pathogen	subject of trial	Number of subjects	Probiotic strain	Pathogen eradication rate	Comments	Reference
*Helicobacter pylori*	Humans	80	*Bifidobacterium bifidum*	↑	Pepsinogen level ↓	Miki et al., 2007 [176]
		14	*Lactobacillus acidophilus*	↑	6 out of 14 *H. pylori* was eradicated	Mrda et al., 1998 [177]
		52	*L. acidophilus* (*johnsonii*) La1	↑	Antrum (14 out of 25), Corpus (11 out of 25)	Felley et al., 2001 [178]
		70	*L. acidophilus* La5 and *Bifidobacterium lactis* Bb12	↑		Wang et al., 2004 [179]
		12	*L. acidophilus*	↑		Gotteland & Cruchet, 2003 [180]
		22	*Lactobacillus brevis* CD2	↑		Linsalata et al., 2004 [181]
		20	*Lactobacillus casei* Shirota	↑		Cats et al., 2003 [182]
		271	*Lactobacillus johnsonii* La1	↑		Gotteland et al., 2008 [183]
		40	*Lactobacillus reuteri* ATCC 55730	↑		Francavilla et al., 2008 [184]
		33	*L. reuteri* SD2112	↑		Imase et al., 2005 [185]
		326	*L. acidophilus* La1	↑		Cruchet et al., 2003 [186]
*Salmonella sp.*	Pigs	8	*Bacillus* based probiotic*	↑		Grandmont et al., 2024 [187]
	Broilers	180	*Bacillus amyloliquefaciens*	↑		Arshad Iqbal et al., 2022 [188]
			**Prebiotic type**			
*Salmonella typhimurium*	Mice	-	GOS mixture	↑		Searle et al., 2009 [189]
	Broilers	-	Refined FOS	↑		Chambers et al., 1997 [190]
*Salmonella enteritidis*	Rats	-	Lactulose	↑		Bovee-Oudenhoven et al., 1997 [191]

^*^PRO-P2702 is a Bacillus strains produced from NUVAC Eco-Sciences (Valcourt, QC, Canada).

‘Biotic therapies present effective strategies for addressing pathogenic infections by promoting beneficial microbiota, strengthening gut barrier function, and modulating immune responses. These approaches are becoming increasingly popular as complementary or alternative treatments to traditional antibiotics and are particularly important in light of the growing issue of antibiotic resistance.

## Conclusion

This review has delved into the complex interactions within the gut microbiota and its profound impact on human health, and the potential of various ‘biotic approaches to restore a healthy gut ecosystem. There are clear connections between diet, pathogenic bacteria, and the composition of the gut microbiota. Distinct dietary components play key roles in shaping the gut microbiota and supporting a diverse microbial ecosystem, whereas inter-individual differences in the microbial composition results in different responses to the same diets. Both chronic disease and the presence of enteropathogens can disrupt the normal composition of the gut microbiota, leading to a state of dysbiosis. Frequently such imbalances are manifested by a decreased prevalence of beneficial bacteria and an increase in potentially harmful species. Interventions with probiotics, prebiotics, synbiotics, and postbiotics are promising ‘biotic therapies to actively shape and maintain a health-promoting gut microbiota. However, more research is needed to better understand the specific mechanisms involved and, in particular, to confirm potential health benefits associated with new, previously un-investigated, members of each ‘biotic family.

As we continue to unravel the complexities of the gut microbiota, it becomes clear that maintaining a balanced microbial gut ecosystem has enormous potential for preventive and therapeutic advances for general human health. This knowledge will contribute to the development of possible new ‘biotic therapies as a new era in health and wellness.

## Data Availability

There are no primary data associated with this review article.
